# The Perfect Storm: A Case of Rapid-Onset Obesity With Hypoventilation, Hypothalamic, Autonomic Dysregulation, Neuroendocrine Tumor (ROHHADNET) With Heart Failure, Narcolepsy, and a Rare Location of a Pelvic Neuroendocrine Tumor

**DOI:** 10.7759/cureus.50341

**Published:** 2023-12-11

**Authors:** Paul Roby, Gretta Smith Beltran, Casey Finch, Sonal Malhotra, Krista Reiling, Ehab Dayyat, Krista Birkemeier, Muppala Raju, Colleen Macmurdo, Edwin Hernandez, Malvika Sagar

**Affiliations:** 1 Internal Medicine, Baylor Scott & White Health, Temple, USA; 2 Pediatrics, Baylor Scott & White Health, Temple, USA; 3 Pediatrics, Baylor College of Medicine, Houston, USA; 4 Pediatrics, Texas Children's Hospital, Houston, USA; 5 Pediatric Neurology, Baylor Scott & White Health, Temple, USA; 6 Radiology, Baylor Scott & White Health, Temple, USA; 7 Neonatology, Baylor Scott & White Health, Temple, USA; 8 Genetics, Baylor Scott & White Health, Temple, USA; 9 Pediatric Pulmonary, Baylor Scott & White Health, Temple, USA

**Keywords:** rohhad, neural crest tumor, autonomic dysregulation syndrome, hypothalamic dysfunction, rapid-onset obesity with hypoventilation

## Abstract

Rapid-onset obesity with hypothalamic dysfunction, hypoventilation, and autonomic dysregulation (ROHHAD) syndrome is a rare disease of concurrent respiratory dysfunction and autonomic dysregulation with endocrine abnormalities. ROHHADNET includes ROHHAD plus coexisting neuroendocrine tumors (NETs). We describe an eight-year-old boy, who originally presented at four years of age with rapid weight gain and hyperhidrosis and who developed mild obstructive sleep apnea (OSA). His clinical course was eventually complicated by hypoxic respiratory failure requiring admission to the pediatric intensive care unit (PICU). Echocardiogram at that time demonstrated dilated cardiomyopathy left ventricular ejection fraction (LVEF) of 28% at time of admission. His respiratory failure persisted despite average volume-assured pressure support (AVAPS) around the clock leading to tracheostomy placement for cardiopulmonary support. He also demonstrated autonomic instability with multiple pituitary hormone deficiencies. Computed tomography (CT) imaging of the abdomen and pelvis demonstrated a presacral soft tissue mass consistent with a tumor of neural crest origin. Daytime somnolence and confusion progressed and a low cerebrospinal fluid hypocretin level revealed a diagnosis of narcolepsy type 1.

## Introduction

Rapid-onset obesity with hypothalamic dysfunction, hypoventilation, and autonomic dysregulation (ROHHAD) is a rare disease with about 200 cases reported [1]. ROHHADNET has been used to describe patients with ROHHAD and commonly associated neuroendocrine tumors [1]. It appears unexpected in early infancy (ages 1.5-seven years), following a period of relatively normal mental and physical growth. Children are susceptible to the development of severe hypoventilation during sleep, which requires artificial ventilation for life support. In severe cases, hypoventilation can occur both awake and asleep. Rapid weight gain is a key symptom of the condition, which is followed by dysregulation of the hypothalamus, alveolar hypoventilation, and autonomic dysregulation. The etiology of ROHHAD remains unclear despite efforts to find a genetic foundation. Postulations have included neural crest origin and autoimmune pathophysiology, with the hypothalamus and periaqueductal gray matter potentially implicated. This case highlights a rare and complicated clinical course of a patient with ROHHAD complicated with heart failure, a rare location for a neuroendocrine tumor (NET), and narcolepsy type 1.

## Case presentation

**Figure 1:**
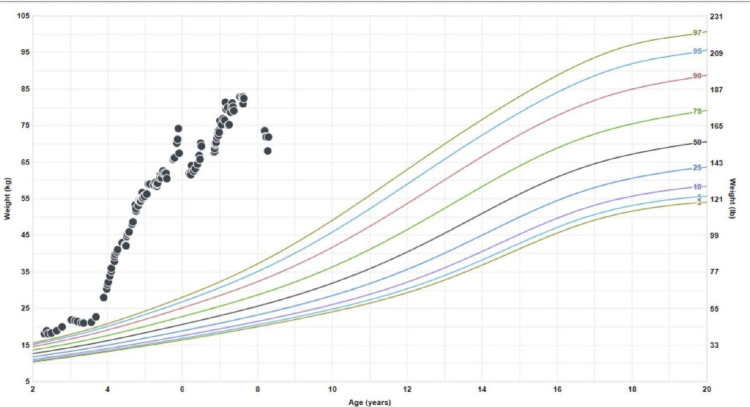
Growth chart demonstrating a rapid increase in weight gain starting just before four years old

The patient presented to the pediatric endocrinology clinic at four years old for evaluation of rapid weight gain of 30 lbs in five months (Figure 1). Associated symptoms included snoring, hyperhidrosis, and hypersomnia. Diagnostic in-lab polysomnogram (PSG) showed mild obstructive sleep apnea (OSA) with an obstructive apnea hypopnea index of 3.7/hour, central apnea index of 1/hour, SpO_2_ nadir of 90%, and maximum transcutaneous CO_2_ of 43 mmHg. Multiple blood gases and PSGs revealed a normal CO_2_ level. Despite several titration polysomnograms (PSGs) with continuous positive airway pressure (CPAP), bi-level positive airway pressure (BPAP) devices, and average volume-assured pressure support (AVAPS), adequate respiratory support could not be attained as AHI was insignificantly changed after titration studies. It was initially thought that the sleepiness was likely due to high CO_2_,_ _and hence different respiratory modalities were tried. During these PSGs, there were several events that did not meet the criteria of hypopneas because they did not result in arousal or hypoxemia. However, during those studies, there was a significant flow limitation throughout the sleep studies. Chromosomal microarray showed a karyotype of arr(1-22)x2,(X,Y)x1. Sequence analysis and deletion/duplication analysis showed that he was heterogenous for variant in the NPR2 gene that is of uncertain clinical significance. Next-generation sequencing (NGS) showed a heterogenous variant in the ALMS1 gene of c.100-105dup, which results in an amino acid substitution of p.Ala34-Ala35dup. He tested negative for PHOX2B variant and Fragile X variants. Leptin levels were 15.4 ng/ml (4.7-23.7 ng/ml). Based on his clinical symptoms and the absence of the PHOX2B variant and other genetic variants, the diagnosis of ROHHAD was presumed despite proven sleep-related hypoventilation.

He was admitted to the PICU for hypoxic respiratory failure at 6.5 years old. Echocardiogram demonstrated dilated cardiomyopathy and severe systolic heart failure with left ventricular ejection fraction (LVEF) of 28% at the time of admission. Echocardiogram seven months prior to that showed no evidence of heart failure. He required AVAPS usage around the clock while still maintaining elevated PCO_2_ levels ranging from 54 to 60 mmHg. A decision was made with the family to perform a tracheostomy for his high need for respiratory support in addition to assisting in his systolic dysfunction. After tracheostomy and initiation of spironolactone, atorvastatin, carvedilol, and lisinopril with nocturnal mechanical ventilation, LVEF improved to 45-50% in 20 months. During this admission, he demonstrated clinical evidence of autonomic instability and multiple pituitary hormone deficiencies, including temperature dysregulation, hypothyroidism, hypercortisolism, and diabetes insipidus. He was placed on bromocriptine, thyroxine, desmopressin, and physiologic hydrocortisone with moderate control of symptoms.

After being treated with invasive ventilatory support, he had normal PCO_2_ when well. His highest recorded PCO_2_ was 72 mmHg on a VBG at seven years old secondary to encephalopathy from fluid electrolyte imbalance.

**Figure 2:**
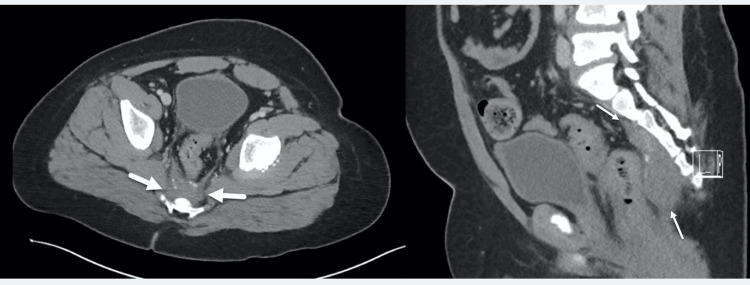
Pelvic CT. Axial (a) and sagittal (b) CT images of the pelvis demonstrate a presacral soft tissue mass (arrows) with high-density calcifications. The abundance of subcutaneous fat density from obesity can also be noted.

Since a neuroendocrine tumor (NET) can occur with ROHHAD, screening CT of the chest, abdomen, and pelvis was performed at seven years old (Figure 2). A 2.9 x 4.2 x 8.6 cm presacral soft tissue mass was identified, consistent with a tumor of neural crest origin. His urinary homovanillic acid (HVA) and vanillylmandelic acid (VMA) were normal at 1.5 mg/L and 0.9 mg/L (0.0-15.0 mg/d and 0.0-7.0 mg/d), respectively. Biopsy and resection were considered high-risk procedures in an asymptomatic case, and conservative management was pursued.

Neurological workup for daytime somnolence and confusion, originally thought to be related to hypoventilation, revealed a diagnosis of narcolepsy type 1 based on a cerebrospinal fluid hypocretin level of <50 pg/ml (>200 pg/ml) at seven years eight months old. He denied cataplexy, hypnagogic or hypnopompic hallucinations, or sleep paralysis.

## Discussion

ROHHAD is a complex disease requiring a multidisciplinary approach. The diagnosis of ROHHAD, with no existing criteria, requires rapid onset obesity in early childhood, alveolar hypoventilation, signs and symptoms of hypothalamic dysfunction and autonomic disturbances, and exclusion of the PHOX2B variant [2] Our patients' heterogenous ALMS1 alanine amino acid substitution has not been previously associated with Alström syndrome (AS) but occurs in an known region of repeat alanines from amine acid 30-36, so it would likely have little significance. Currently, there is no known genetic variant associated with ROHHAD[PR2]. The diagnosis of ROHHAD was challenging as hypercapnia was noted late in the clinical course.

The incidence of NET in the general pediatric population is about 2.8 cases per million, but it occurs in 40-50% of patients with ROHHAD [1]. The most common NET reported in ROHHAD cases is ganglioneuroma with an average age of four years at diagnosis. The common locations for these tumors are intraabdominal, specifically adrenal, or paravertebral [1,3]. The majority of these tumors are benign, but a report of malignant neuroblastoma with ROHHAD necessitates screening [4]. The resection of NET in at least two ROHHAD patients resulted in no symptomatic improvement [5]. In our patient, the differential for his presacral tumor included neuroendocrine tumors, teratoma, and chordoma. Based on his ROHHAD diagnosis, paraspinal location, and no osseous destruction, a NET diagnosis was most likely. His normal range urinary catecholamines are consistent with other case reports [6].

While there are no specific guidelines currently in place for NET screening in ROHHAD patients, several reports have suggested protocols. The atypical location of his presacral tumor suggests expansion of screening into the pelvic region and should be considered for patients diagnosed with ROHHAD. 

Cardiac involvement in ROHHAD is typically limited to arrhythmias, blood pressure dysregulation, and cor pulmonale. One case of cardiomyopathy secondary to a hypoxic event has been reported, which was transient and responded to medical management [7]. The major cause of death in individuals with ROHHAD is thought to be cardiac arrest secondary to respiratory failure [3]. Our patient’s presentation with dilated cardiomyopathy and systolic dysfunction was refractory to medical management. He was not a candidate for ventricular assist device due to low ejection fraction, extracorporeal cardiopulmonary resuscitation, or heart transplant. Given the high respiratory support, inability to tolerate facemask, and his degree of systolic dysfunction, the decision was made to pursue with tracheostomy, especially given that most children with ROHHAD ultimately require tracheostomy [7].

Narcolepsy has rarely been reported with ROHHAD. Only one case with low CSF hypocretin level meeting criteria for narcolepsy type 1 has been reported in a patient with ROHHAD [8]. Our patient had a CSF hypocretin level of <50 pg/ml, leading to a diagnosis of narcolepsy type 1 despite evidence of cataplexy or other symptoms of narcolepsy.

ROHHAD is a rare syndromic cause of childhood obesity with many overlapping symptoms with other childhood obesity syndromes, such as obesity hypoventilation syndrome (OHS), Prader-Willi syndrome (PWS), Bardet-Biedl syndrome (BBS), and Alström syndrome (AS). Obesity hypoventilation syndrome (OHS) is a condition with hypoventilation, BMI >30 kg/m^2^, and sleep-related breathing disorders. Hypoventilation with both OHS and ROHHAD typically follows obesity, but OHS lacks the hypothalamic dysfunction that develops in patients with ROHHAD. PWS is another condition with childhood obesity and many overlapping symptoms.

PWS is a genetic disorder where a paternal copy of the PWS region chromosome 15 is absent or imprinted but silenced. The phenotype of PWS is variable, and it can present early with neonatal hypotonia, poor feeding, a weak cry, and characteristic facial features, such as almond-shaped eyes, thin upper lip, downturned corners of the mouth, and a narrow face. Later developments of PWS including but not limited to hyperphagia, excessive weight gain, hypogonadism, and intellectual disability are common manifestations of the syndrome. Diagnosis of PWS is made with DNA methylation testing with several different implicated genes. An important differentiating feature of the two conditions is that patients with ROHHAD are healthy up to their onset of rapid obesity, often occurring around the age of two.

The other key difference between PWS and ROHHAD is the genetic cause on chromosome 15 in PWS, which is lacking in ROHHAD. BBS is an autosomal recessive ciliopathy with a variable phenotype and obesity often presenting in the first year of life. Common presentations are retinal cone-rod dystrophy, postaxial polydactyly, cognitive impairment, hypogonadism, and kidney disease. Diagnosis is made with a combination of clinical features and NGS with 16 genes associated with >80% of clinically diagnosed BBS. While ROHHAD and BBS share childhood obesity occurring early in child childhood, there are significant difference in other clinical symptoms and BBS having a known genetic cause.

AS is an autosomal recessive disorder caused by variants in the ALMS1 gene on chromosome 2p13, which also presents with childhood obesity. No history of consanguinity in parents was found. Other common symptoms in this variable syndrome are cone-rod retinal dystrophy, sensorineural hearing loss, severe insulin resistance, cardiomyopathy, renal anomalies, and respiratory disease. A combination of the ALMS1 variant and clinical symptoms are used to confirm the diagnosis of AS. Similarities between AS and ROHHAD are limited to childhood obesity and cardiomyopathy, seen uniquely in our patient, and these syndromes can be differentiated based on genetic testing, retinal dystrophy, and sensorineural hearing loss in addition to symptoms appearing early in infancy that need to be addressed early with patient counselling and early interventional therapies available.

## Conclusions

This illustrative case highlights the challenges associated with diagnosing ROHHAD and a novel clinical course including cardiac failure, pelvic NET, and narcolepsy type 1. This case is also useful to consider the differential diagnosis for syndromic causes of childhood obesity.

Sadly, several months later, he was admitted for abdominal pain and hypoxia. He was found to be in septic shock and later passed away peacefully with family at his side.
